# Isoflurane Exposure during Mid-Adulthood Attenuates Age-Related Spatial Memory Impairment in APP/PS1 Transgenic Mice

**DOI:** 10.1371/journal.pone.0050172

**Published:** 2012-11-19

**Authors:** Diansan Su, Yanxing Zhao, Huan Xu, Beilei Wang, Xuemei Chen, Jie Chen, Xiangrui Wang

**Affiliations:** Department of Anesthesiology, Renji Hospital, School of Medicine, Shanghai Jiaotong University, Shanghai, China; Oregon Health & Science University, United States of America

## Abstract

Many in vitro findings suggest that isoflurane exposure might accelerate the process of Alzheimer Disease (AD); however, no behavioral evidence exists to support this theory. In the present study, we hypothesized that exposure of APP/PS1 transgenic mice to isoflurane during mid-adulthood, which is the pre-symptomatic phase of amyloid beta (Abeta) deposition, would alter the progression of AD. Seven-month-old Tg(APPswe,PSEN1dE9)85Dbo/J transgenic mice and their wild-type littermates were exposed to 1.1% isoflurane for 2 hours per day for 5 days. Learning and memory ability was tested 48 hours and 5 months following isoflurane exposure using the Morris Water Maze and Y maze, respectively. Abeta deposition and oligomers in the hippocampus were measured by immunohistochemistry or Elisa 5 months following isoflurane exposure. We found that the performance of both the transgenic and wild-type mice in the Morris Water Maze significantly improved 48 hours following isoflurane exposure. The transgenic mice made significantly fewer discrimination errors in the Y maze following isoflurane exposure, and no differences were found between wild-type littermates 5 months following isoflurane exposure. For the transgenic mice, the Abeta plaque and oligomers in the hippocampus was significantly decreased in the 5 months following isoflurane exposure. In summary, repeated isoflurane exposure during the pre-symptomatic phase not only improved spatial memory in both the APP/PS1 transgenic and wild-type mice shortly after the exposure but also prevented age-related decline in learning and memory and attenuated the Abeta plaque and oligomers in the hippocampus of transgenic mice.

## Introduction

Inhaled anesthetics are used for general anesthesia in thousands of surgeries each day worldwide. However, there has been increasing concern about their neurotoxic effects, especially isoflurane, because these anesthetics may be involved in postoperative cognitive dysfunction (POCD).

An initial study by Eckenhoff showed that isoflurane increased the aggregation of the amyloid beta (Abeta) protein and induced cytotoxicity [1]. Another study from the same group reported that isoflurane enhanced the small oligomers of Abeta [2]. The Xie study showed that isoflurane induces apoptosis, alters the processing of amyloid precursor protein (APP), and increases the production of Abeta in a human neuroglioma cell line [3]. Because the deposition of Abeta is a key event in the pathogenesis of Alzheimer’s disease (AD), these in vitro findings suggest that isoflurane exposure might worsen the symptoms or accelerate the process of AD. However, Bianchi’s findings [4] suggest otherwise. No decrease in learning and memory and no increase in Abeta deposition following isoflurane exposure were found in 12-month-old Tg2576 transgenic mice. However, the 12-month-old mice may have been too old; thus, their learning and memory may have already been considerably compromised. Many patients in the pre-symptomatic phase of AD are exposed to anesthesia and undergo surgery for different reasons. In the present study, we investigated whether isoflurane exposure during mid-adulthood, which is the pre-symptomatic phase in which Abeta deposition begins, alters the progression of AD. APP/PS1 transgenic mice and their non-transgenic wild-type littermates were exposed to isoflurane at 7 months of age, and behavioral changes were tested 48 hours and 5 months later. Finally, the Abeta plaque and oligomer was tested 5 months following the isoflurane exposure.

## Results

### Isoflurane Exposure Improved the Short-term Spatial Memory of Both APP/PS1 Transgenic and Wild-type Mice

**Figure 1:**
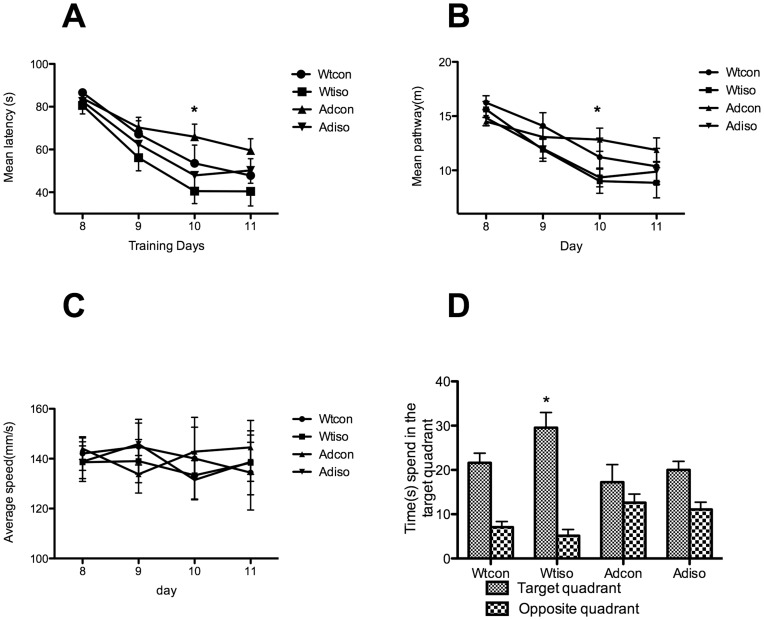
The Morris Water Maze was performed 48 hours after the final isoflurane exposure. The isoflurane exposure, but not the presence of the transgene, significantly affected the escape latency, swimming pathway and the swimming time in the target quadrant during probe test. The average swimming speeds were similar among groups. A: The mean latency to find the platform was shorter following the isoflurane exposure for both the transgenic and wild-type mice. *: p<0.05 on the third day compared with transgenic and wild-type mice not exposed to isoflurane. B: The mean pathway to find the platform was shorter following the isoflurane exposure for both the transgenic and wild-type mice. *: p<0.05 on the third day compared with transgenic and wild-type mice not exposed to isoflurane. C: The swimming speed was similar among the four groups. D: During the probe trial, wild-type mice exposed to isoflurane spent more time in the target quadrant following the isoflurane exposure. The X-axis (days) corresponds to the testing paradigm in Figure 1 (days 8–11). (Ad-iso, n = 20; Ad-con, n = 20; Wt-iso, n = 16; Wt-con, n = 13).

To evaluate the short-term spatial memory effects of isoflurane on the APP/PS1 transgenic (Tg) and wild-type (Wt) mice, Morris Water Maze(MWM) was performed 48 hours following the isoflurane exposure. As shown in Figure 1A, the reference memory of all animals improved as training progressed. The statistical results indicate that isoflurane had a significant effect on the escape latency (p = 0.023). However, the transgene had no significant effects on the escape latency (p = 0.122). There was no interaction between the isoflurane exposure and the presence of the transgene (p = 0.983). With the UNIANOVA, the latency to find the platform for mice exposed to isoflurane was shorter than in the control mice at day 3, both in the transgenic mice (p = 0.037) and their wild-type littermates (p = 0.035). Similar results were found for the mean pathway (Fig. 1B). Isoflurane exposure significantly affected the pathway (p = 0.024), but the transgene did not (p = 0.853). No interaction between the isoflurane exposure and the presence of the transgene was found (p = 0.950). With the UNIANOVA, the pathway was shorter on day 3 following the isoflurane exposure both in the transgenic mice (p = 0.025) and their wild-type littermates (p = 0.020). The swimming speed was similar among the different groups, and none of the three factors (i.e., isoflurane exposure, transgene or time) affected swimming speed significantly (p>0.150) (Fig. 1C).

A probe test was performed on the second day after the final reference memory testing to further test the retention of memory in the mice. The statistical results showed that the isoflurane exposure significantly affected the swimming time in the target quadrant (p = 0.035). However, the presence of the transgene did not significantly affect the swimming time in the target quadrant (p = 0.143). There was no interaction between the two factors (isoflurane and transgene, p>0.397). With the UNIANOVA, the time spent swimming in the target quadrant was longer following the isoflurane exposure in the wild-type mice (p = 0.034), but no statistical significance was found following isoflurane exposure in the transgenic mice (p = 0.389) (Fig. 1D; Ad-iso, (Female/Male); n = 20(6/14); Ad-con, n = 20(8/12); Wt-iso, n = 16(5/11); Wt-con, n = 13(5/8)).

### Isoflurane Exposure during Mid-adulthood Attenuated the Learning and Memory Impairment of Aged APP/PS1 Transgenic Mice

**Figure 2:**
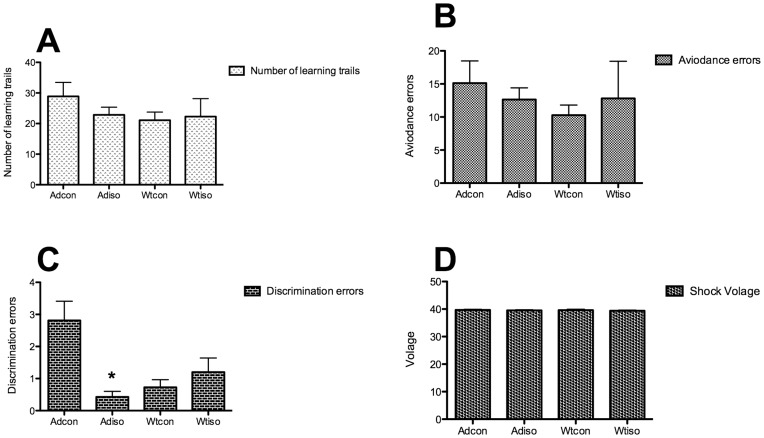
The Y maze was performed five months following the isoflurane exposure. A: The number of learning trails to reach the final criterion. No difference was found among the groups. B: Avoidance errors. No difference was found among the groups. C: Discrimination errors. The mice in the Ad-iso group made significantly fewer discrimination errors than the mice in the Ad-con group. No difference was found between the two wild-type groups. *: p<0.05 compared with the Ad-con group. D: No difference in the foot shock voltage was found among the groups. (Ad-iso, n = 20; Ad-con, n = 20; Wt-iso, n = 16; Wt-con, n = 13).

To evaluate the long-term effects of isoflurane exposure during mid-adulthood on the learning and memory of aged mice, the Y-maze test was performed five months following exposure. A three-way ANOVA was conducted to test the effects of isoflurane exposure, the presence of the transgene and gender on the learning and memory of aged mice. There was no statistical difference among the groups in terms of total number of trials to achieve task completion (p>0.45; Fig. 2A). Similarly, for the active avoidance errors, no statistical difference was found among the groups (p>0.45; Fig. 2B). For the discrimination errors, there was a significant interaction between the effects of the isoflurane exposure and the presence of the transgene (p = 0.012; Fig. 2C). The isoflurane exposure significantly decreased the discrimination errors (p = 0.036), but gender (p = 0.957) and the presence of the transgene (p = 0.244) did not affect these errors. The simple main effects analysis showed that transgenic mice made fewer discrimination errors following isoflurane exposure (p = 0.001), but the wild-type mice did not (p = 0.483). There was no difference in the level of foot shock among the groups (Fig. 2D).

### Isoflurane Exposure during Mid-adulthood Decreased the Abeta Plaque Deposition and Oligomers of the Aged Transgenic Mice

**Figure 3:**
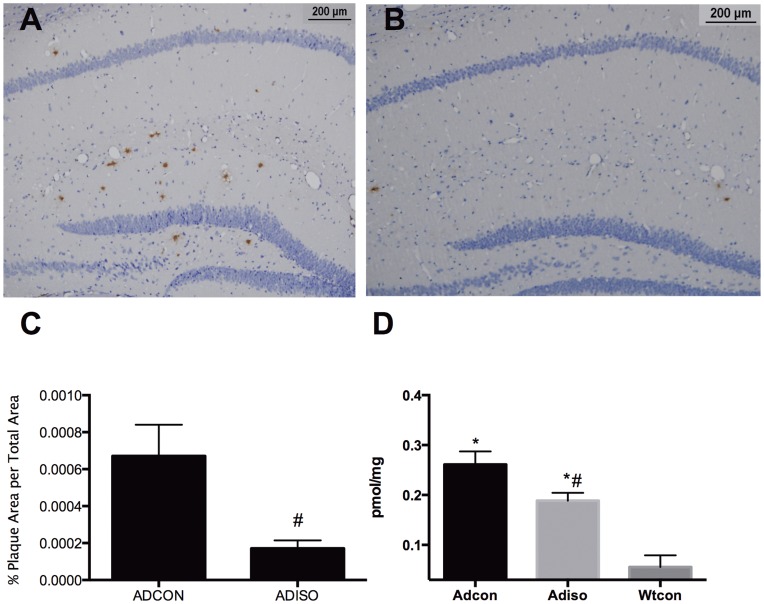
Abeta plaques and oligomers in the hippocampus. Following the isoflurane exposure during mid-adulthood, compared to the Ad-con group (A), the Abeta plaque was significantly decreased in the Ad-iso group (B). Statistical analysis of the percent area of the Abeta plaques in the hippocampus and dentate gyrus(C). Compared to the Ad-con group, the Abeta oligomer was significantly decreased in the Ad-iso group (D) 5 months following isoflurane exposure. (n = 5).

Many Abeta plaques were found in the transgenic mice at 12 months of age (Fig. 3A). The total area of Abeta plaques in the hippocampus and dentate gyrus (Fig. 3B) was significantly decreased in the animals exposed to isoflurane compared with the controls (p = 0.02; Fig. 3C). No plaques were found in the wild-type mice, as expected. Compared with the wild-type mice, Abeta oligomer in the hippocampus (Fig. 3D) was significantly higher in the transgenic mice (p<0.0001). Between the two transgenic group, Abeta oligomer decreased significantly in the animals with isoflurane exposure compared with the control (p = 0.041).

### Hemodynamics and Blood Gas Analysis

**Figure 4:**
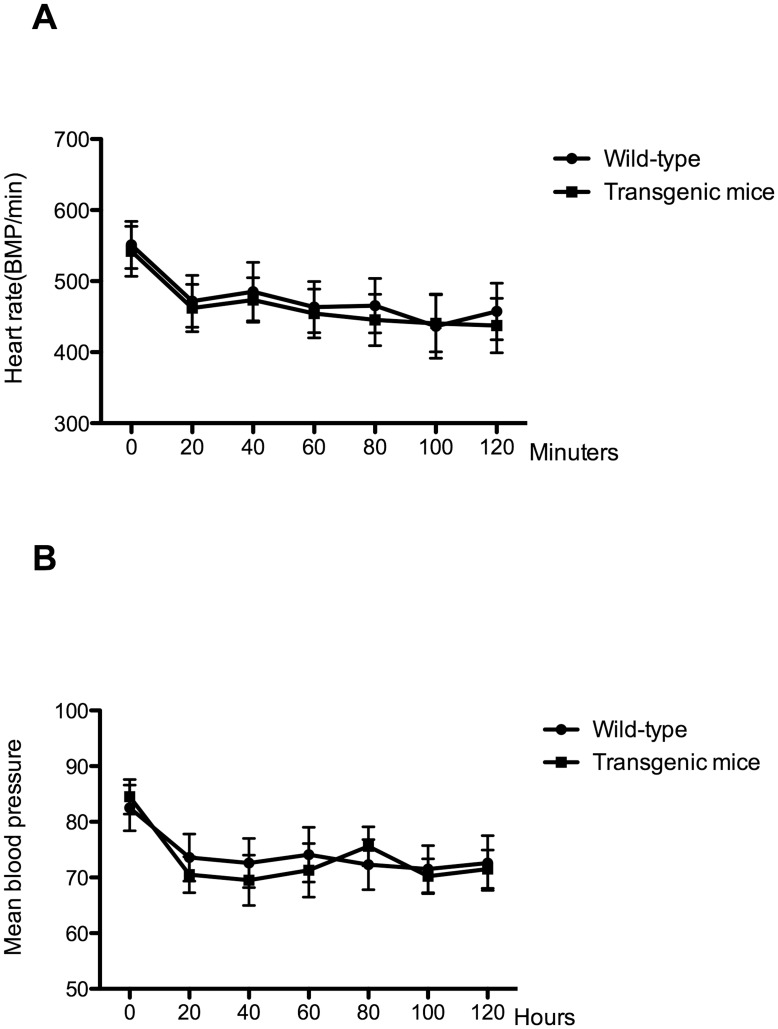
A two-hour isoflurane exposure did not significantly affect blood pressure and heart rate. Animals maintained stable heart rates (A) and blood pressure (B). (n = 5).

Isoflurane exposure may decrease blood pressure and depress respiration; therefore, we monitored blood pressure and heart rate during isoflurane anesthesia. As shown in Figure 4, blood pressure and heart rate decreased following isoflurane exposure in both the transgenic and wild-type mice, but these parameters remained stable over the course of the anesthesia. To prevent hypoxia during isoflurane exposure, we analyzed the blood gas levels at the end of isoflurane exposure, which revealed normal oxygenation (transgenic mice/wild type: 128.67±1.76 mmHg/130.56± 2.13 mmHg), adequate PCO_2_ values (41.46±1.43 mmHg/42.23± 1.56 mmHg) and no acidosis (pH: 7.35±0.06/7.37±0.05).

## Discussion

In this study, we demonstrated that spatial memory improved 48 hours after isoflurane exposure in 7-month-old APP/PS1 transgenic mice and their non-transgenic littermates. Additionally, isoflurane exposure during mid-adulthood attenuated learning and memory deficits and decreased the deposition of Abeta plaque and oligomers 5 months later in the APP/PS1 transgenic mice.

The effect of isoflurane on the spatial memory of adult animals is still controversial. In a study by Culley [5], rats were initially trained in the 12-arm maze and then treated with 1.2% isoflurane and 70% nitrous oxide/30% oxygen for 2 h. They found that anesthetized 6-month-old rats made fewer errors 8 weeks following anesthesia. Crosby [6] demonstrated that 1.2% isoflurane and 70% nitrous oxide administered for 2 hours improved 12-arm maze performance 2 weeks later. Rammes [7] showed that 1.33% isoflurane anesthesia induced an improvement in learning in the hole board test. In our previous study, we also demonstrated that repeated isoflurane exposure improved the spatial memory of 2-month-old mice [8]. However, Butterfield [9] demonstrated that single or repeated isoflurane exposure did not affect spatial memory in adult mice. Culley [10] anesthetized 6-month-old rats using 1.2% isoflurane and 70% nitrous oxide/30% oxygen for 2 h and found impaired learning and memory in the 12-arm maze. In the present study, 7-month-old APP/PS1 transgenic mice and their non-transgenic littermates were repeatedly exposed to isoflurane. These transgenic mice begin to produce Abeta plaques at 7 months of age, and they demonstrated no behavioral changes until 12 months of age [11]. Therefore, it is reasonable to suggest that the improved MWM performance following isoflurane exposure in both the transgenic and non-transgenic mice was due to the fact that the transgene did not significantly affect MWM performance. Moreover, our current results are consistent with our recent findings showing that learning and memory improve in C57 mice 48 hours following isoflurane exposure [8].

The objective of this study was to observe if isoflurane exposure during the pre-symptomatic phase alters the progression of AD. Five months after mice were exposed to isoflurane, their behavioral changes were tested using the Y-maze. For the wild-type mice, there was no difference on the performance in Y-maze after isoflurane exposure, which is consistent with our previous study [8] in which the improved spatial memory of adult mice repeatedly exposed to isoflurane was temporary because no improvements were found two weeks later [8]. The interesting finding in this study is that transgenic mice exposed to isoflurane made significantly fewer discrimination errors in the Y-maze compared with the controls. This finding indicates that the isoflurane exposure during mid-adulthood improved the age-related learning and memory deficits in transgenic mice, which normally lead to neurocognitive dysfunction. These results show that the isoflurane exposure during mid-adult not only improved the behavior and decreased Abeta plaque deposition in the APP/PS1 transgenic mice, but exposure also had very long-term protective effects. Tang [12] showed similar results with halothane: the learning and memory of 2-month-old triple-transgenic AD mice improved with 1 MAC halothane.

However, Tang [12] did not find any changes in the behavior or amyloid plaque deposition level 2 months after exposure to 1 MAC isoflurane in the 2-, 4- or 6-month-old triple-transgenic AD mice. The present study has many differences from the Tang study. First, we used APP/PS1 transgenic mice and not triple-transgenic mice. This difference in the genetic background may lead to different results. Second, although we used a similar concentration of isoflurane (1.1%), this amount of isoflurane was at a sub-MAC concentration in the APP/PS1. Finally, the procedures for the isoflurane exposure and behavior tests were different. The mice in the present study were exposed to isoflurane for 2 hours for 5 days, not for 5 hours once a week for 4 weeks [12]. Additionally, the behavioral changes were tested 48 hours and 5 months later rather than 2 months later [12]. Mice of both genders were used in the present study; however, gender did not significantly affect the learning and memory tests.

Perucho [13] also used young transgenic AD mice but obtained different results: APPswe mice had increased mortality, lower responsiveness and an increased number of apoptotic cells after isoflurane (2%) exposure for 20 minutes [13]. The concentration of isoflurane used in the present study was relatively lower (1.1%), which might explain the contradictory results.

Several studies have shown that the ultimate effects of isoflurane critically depend on both the concentration and duration of exposure. Pan [14] demonstrated that treatment with 0.5% isoflurane for 8 hours attenuated the hypoxia-induced activation of caspase-3 and the levels of the aspartyl protease β-site amyloid precursor protein-cleaving enzyme in H4 human neuroglioma cells; treatment with 2% isoflurane for 8 hours enhanced this response. Wei [15] reported that pre-conditioning with isoflurane for one hour dose-dependently inhibited the neurotoxicity induced by a 24-h exposure to 2.4% isoflurane in both primary cortical neurons and PC12 cells. Lee [16] showed that 2% isoflurane post-conditioning for 20 or 30 minutes after oxygen-glucose deprivation ameliorated the cell injury induced thereof. However, exposure to 2% isoflurane for 60 minutes or 2.5% isoflurane for 20–30 minutes had no post-conditioning effects. These results suggest that the effects of isoflurane may be concentration- and duration-dependent. In the present study, the concentration of isoflurane was relatively low, and the duration was relatively short. Although no study has used isoflurane for 2 hours for 5 days as pre-conditioning, many studies have demonstrated that chronic ischemia or pharmacological pre-conditioning is associated with a reduction in myocardial infarct size [17], [18]. Many other studies demonstrated the neuroprotective effects of isoflurane preconditioning against ischemia [19], [20] or hypoxia [21], [22]. Therefore, pre-conditioning effects may be involved.

In cell culture models, isoflurane alters APP processing and increases the production of Abeta [3], [23]; however, in the present study, we found that isoflurane exposure decreased the Abeta plaque in the hippocampus. However, the Abeat plaques is poorly correlated with the synaptic loss or cognitive impairment in AD subjects [24]. At the same time, accumulating evidences demonstrated that there are a strong correlation between the Abeta oligomer and the synaptic loss and cognitive impairment[24]–[27]. In present study, we found that the Abeta oligomers reduced significantly after isoflurane exposure. However, further studies are required to elucidate the detailed mechanism by which this occurs.

Our study has many limitations. The MWM and Y-maze studies were performed at different time points. The MWM primarily measures hippocampal function, whereas the Y-maze measures a mixture of hippocampal and amygdala function. We did not use both tests at both time points. Although we assumed the protective effects of isoflurane found in the present study were due to pre-conditioning, we did not confirm this. Therefore, more studies are warranted.

In conclusion, isoflurane exposure during mid-adulthood improved not only the spatial memory of both the APP/PS1 transgenic and wild-type mice but also the impaired learning and memory in aged transgenic mice and attenuated the Abeta plaque and oligomers in the hippocampus.

## Materials and Methods

### Animals

This protocol was approved by the Shanghai Jiaotong University, School of Medicine, Animal Care and Use Committee (Permit Number: Renji-09-1013). All procedures were performed in accordance with the National Institutes of Health (NIH) guidelines for animal care (Guide for the Care and Use of Laboratory Animals, Department of Health and Human Services, NIH Publication No. 86–23, revised 1985).

Seven-month-old male and female APP/PS1 transgenic mice and wild-type littermates were used in this study. The mice were bred by mating B6C3-Tg(APPswe,PSEN1dE9)85Dbo/J male with F1 female C57 and C3H mice.

This mouse strain is a double transgenic hemizygote that expresses a chimeric mouse/human amyloid precursor protein (Mo/HuAPP695swe) and mutant human presenilin 1 (PS1-dE9). Abeta deposits in the brain can be detected by 6 to 7 months of age. All animals were bred in the animal facilities at the School of Medicine, Shanghai Jiaotong University. The initial pairs of mice were a gift from Prof. Shumin Duan, Shanghai Institutes for Biological Science, Chinese Academy of Sciences. Genetic phenotyping of tail samples was performed by proteinase K digestion, DNA extraction and PCR analysis.

### Isoflurane Exposure

**Figure 5:**
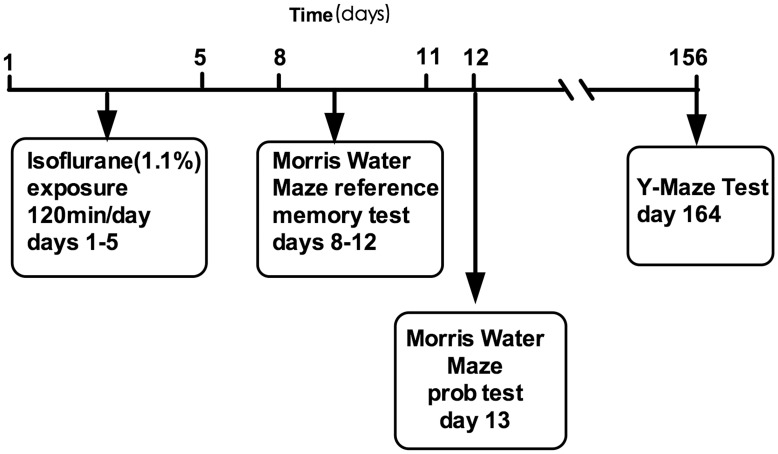
Schematic timeline of the experimental paradigm.

Both the APP/PS1 transgenic and wild-type mice were exposed to 1.1% isoflurane in a chamber that was partially submerged in a circulating 37°C water bath. Thirty percent oxygen and 70% nitrogen flowed at a rate of 2 L/min through a calibrated isoflurane vaporizer into the chamber. The concentration of oxygen, carbon dioxide and isoflurane was continually monitored in the effluent chamber gas using infrared absorbance (Ohmeda 5330, Detex-Ohmeda, Louisville, CO). Mice were exposed to isoflurane for 2 hours per day for 5 days (a total of five exposures) (Fig. 5, days 1–5). Mice breathed spontaneously and easily without any support during the periods of isoflurane exposure. The mice in the control groups were exposed to vehicle gas (30% O_2_+70% N_2_) for 2 hours per day for 5 days. Rectal temperatures were measured after each isoflurane exposure, and no significant changes were observed (Transgenic mice/wild-type: 37.6±0.3°C/37.5±0.4°C). All animals recovered completely within 10 minutes following the isoflurane exposure, and no animals died.

### Hemodynamic Monitoring and Blood Gas Analysis

Isoflurane (1.1%) anesthesia was administered to both wild-type and transgenic mice for 2 hours via a mask specifically designed for mice (n = 5). The blood pressure and heart rate were measured before and during anesthesia (every 20 minutes) with a noninvasive blood pressure meter (Softron, Beijing, China). At the end of the anesthesia, the abdomen of each animal was quickly opened, and the abdominal aorta was exposed. With a 24 gauge venous catheter, 0.5–1 ml of blood was drawn for the blood gas analysis.

### Morris Water Maze

To identify short-term behavioral changes two days after isoflurane exposure (Fig. 5, days 8–13), the Morris Water Maze (MWM) was performed by all animals. The test was administered by an operator blinded to the group conditions. The MWM consisted of a painted circular pool (110 cm in diameter and 30 cm in depth) in which mice were trained to escape from the water by swimming to a hidden platform 1.5 cm beneath the surface, the location of which could only be identified by using distal extra-maze cues attached to the room walls. The water was kept at 20°C and made opaque with titanium dioxide throughout all training and testing. The pool was divided into four quadrants: north (Target), south (Opposite), east (Adjacent 1) and west (Adjacent 2). The experiments were recorded using a camera connected to a video recorder and a computerized tracking system.

The MWM testing began on the 8^th^ day from the beginning of isoflurane exposure and continued for 5 days. The first 4 days (Fig. 5, days 8–11) were the reference memory test phase, which consisted of 16 training trials: 4 training trials per day for 4 training days with an inter-trial interval of 30–40 min. At the beginning of each trial, the mouse was placed into one of four quadrants facing the wall. Although the starting point was randomly selected, the protocol was fixed at the beginning of each trial and was maintained throughout all 4 trials. Each mouse was given 90 s to find and mount the platform. Thirty seconds after the mouse mounted the platform, the mouse was removed, placed in a holding cage and warmed with a heating lamp. The mice that failed to locate and mount the platform within 90 s were gently guided to the platform and required to remain there for 30 s before they were transferred to the holding cage. A video camera mounted above the pool was used to track the mice. The amount of time spent finding and mounting the platform (escape latency), the swimming pathway before finding the platform and the swimming speed were calculated from the recorded videos using MWM software (Shanghai Jiliang Software Technology Co. Ltd., China).

On the fifth day (Fig. 5, day 12), a probe test was performed in which we removed the platform, and animals were allowed to swim freely for 60 s. The amounts of time spent in the target quadrant and the opposite quadrant were recorded.

### Y-maze

Five months later (Fig. 5 day 156), the avoidance learning task was performed as previously described [28] in a Y-shaped black apparatus with three identical arms, each 22 cm long, 6.5 cm wide, and 10 cm high. The floor was equipped with electric grids controlled by a computer, and a camera on the top of the Y-maze recorded and provided the position of the animals to the computer. Among the three arms, one was defined as the ‘start arm’, one was the ‘wrong arm’ and the third was the ‘correct arm’. Animals had to leave the ‘start arm’ within 5 seconds and escape into the ‘correct arm’ to avoid foot shocks. Active avoidance errors were recorded if the animals did not leave the ‘start arm’ within 5 seconds. If the mouse chose the ‘wrong arm’, a discrimination error was recorded. Foot shocks were administered each 7 seconds until the animals chose the ‘correct arm’. The foot shock level was changed individually (maximum: 40 V) according to the performance of the mouse in the first trial or until the mouse suddenly lifted one or two paws from the grid at the bottom of the Y-maze after the shock. One trial per minute was performed until the mouse reached the final criterion of correctly performing seven out of eight consecutive trials. The Y-maze was cleaned between each mouse to avoid odor confounding. The total trials, active avoidance errors and discrimination errors were recorded.

### Immunohistochemistry

Following the Y-maze test, all animals were briefly anesthetized with isoflurane and perfused via the left ventricle with normal saline (4°C) and paraformaldehyde (4%). The brains were removed and embedded in paraffin in order to perform immunohistochemistry for Abeta. Six coronal 4- to 5 µm sections from each mouse (n = 5), each separated by approximately 100 µm intervals starting 1.6 mm posterior to the bregma and extending into the caudal region, were used for the immunohistochemical staining with DAB. Immunohistochemistry was performed as previously described, [4] and the concentration of primary Abeta antibody was 1∶50 (Vector Labs, Cat. No. VP-B203). All the sections were photographed with a microscope (BX51, Olympus) equipped with a digital camera (DF71, Olympus). The area of Abeta plaques in the hippocampus and dentate gyrus was calculated with ImageJ (free software from NIH) and special plug-ins (http://www.uhnres.utoronto.ca/facilities/wcif/fdownload.html).

A researcher blinded to the group conditions performed the quantification of the Abeta immunostaining. The threshold color function was used to set a threshold, which included all positive particles and no background contamination. Using the particle analysis function, the percent area of Abeta plaques in the hippocampus and dentate gyrus was calculated.

### Abeat Oligomers Measurement

The Abeat oligomers in hippocampus was quantified by ELISA(82E1-specific Kit, IBL America, Minneapolis, MN,USA). Mouse hippocampus were dissected and were homogenized in extraction buffer consisting of 50mM Tris (pH 7.4), 2 mM EDTA, 400 mM NaCl, and complete protease inhibitor cocktail (Roche). The homogenates were centrifuged at 1,200 g for 15 min at 4°C and supernatants were analyzed for Abeat oligomers according to the manual of the kit. Protein concentrations of all samples were measured using a BCA protein assay kit (Pierce, Thermo Fisher Scientific, Rockford, IL USA). Abeat oligomers were expressed as pmol/mg of protein.

### Statistics

All the data were presented as the mean±SEM. Statistical Package for the Social Sciences (SPSS) v.10.0 was used for the statistical analyses. A three-way ANOVA with repeated measures (i.e., isoflurane exposure and transgene as two factors between subjects, time as a repeated measures factor) was used to analyze the water maze escape latency, mean pathway and average speed. UNIANOVAs were used to test the simple main effects of grouping the variables at each time point. A two-way ANOVA (with isoflurane and the transgene as the two variables) was used for the probe quadrant trial data. A three-way ANOVA (i.e., isoflurane, the transgene and gender) was used for the Y maze data. UNIANOVAs were used to test the simple main effects for the significant interaction factors. An independent t test was used for the percent area occupied by Abeta plaques. One-way ANOVA was used for the Abeta oligomers and following with post hoc by LSD. Differences were considered to be statistically significant at p<0.05.
